# Radiomic nomogram based on MRI to predict grade of branching type intraductal papillary mucinous neoplasms of the pancreas: a multicenter study

**DOI:** 10.1186/s40644-021-00395-6

**Published:** 2021-03-09

**Authors:** Sijia Cui, Tianyu Tang, Qiuming Su, Yajie Wang, Zhenyu Shu, Wei Yang, Xiangyang Gong

**Affiliations:** 1 Department of Radiology, Zhejiang Provincial People’s Hospital, Affiliated People’s Hospital of Hangzhou Medical College, 158 Shangtang Road, Hangzhou, 310000 China; 2 The Second Clinical Medical College, Zhejiang Chinese Medical University, Hangzhou, 310053 China; 3 Department of Hepatobiliary and Pancreatic Surgery, the First Affiliated Hospital, Zhejiang University School of Medicine, Hangzhou, China; 4 Zhejiang Provincial Key Laboratory of Pancreatic Disease, Hangzhou, China; 5 Department of General Surgery, The Second Affiliated Hospital of Zhejiang University School of Medicine, Hangzhou, Zhejiang China; 6 Bengbu Medical College, Bengbu, 233000 China; 7 Institute of Artificial Intelligence and Remote Imaging, Hangzhou Medical College, Hangzhou, China

**Keywords:** Branch duct type, Intraductal papillary mucinous neoplasm, MRI, Radiomics, Nomogram

## Abstract

**Background:**

Accurate diagnosis of high-grade branching type intraductal papillary mucinous neoplasms (BD-IPMNs) is challenging in clinical setting. We aimed to construct and validate a nomogram combining clinical characteristics and radiomic features for the preoperative prediction of low and high-grade in BD-IPMNs.

**Methods:**

Two hundred and two patients from three medical centers were enrolled. The high-grade BD-IPMN group comprised patients with high-grade dysplasia and invasive carcinoma in BD-IPMN (*n* = 50). The training cohort comprised patients from the first medical center (*n* = 103), and the external independent validation cohorts comprised patients from the second and third medical centers (*n* = 48 and 51). Within 3 months prior to surgery, all patients were subjected to magnetic resonance examination. The volume of interest was delineated on T1-weighted (T1-w) imaging, T2-weighted (T2-w) imaging, and contrast-enhanced T1-weighted (CET1-w) imaging, respectively, on each tumor slice. Quantitative image features were extracted using MITK software (G.E.). The Mann-Whitney U test or independent-sample t-test, and LASSO regression, were applied for data dimension reduction, after which a radiomic signature was constructed for grade assessment. Based on the training cohort, we developed a combined nomogram model incorporating clinical variables and the radiomic signature. Decision curve analysis (DCA), a receiver operating characteristic curve (ROC), a calibration curve, and the area under the ROC curve (AUC) were used to evaluate the utility of the constructed model based on the external independent validation cohorts.

**Results:**

To predict tumor grade, we developed a nine-feature-combined radiomic signature. For the radiomic signature, the AUC values of high-grade disease were 0.836 in the training cohort, 0.811 in external validation cohort 1, and 0.822 in external validation cohort 2. The CA19–9 level and main pancreatic duct size were identified as independent parameters of high-grade of BD-IPMNs using multivariate logistic regression analysis. The CA19–9 level and main pancreatic duct size were then used to construct the radiomic nomogram. Using the radiomic nomogram, the high-grade disease-associated AUC values were 0.903 (training cohort), 0.884 (external validation cohort 1), and 0.876 (external validation cohort 2). The clinical utility of the developed nomogram was verified using the calibration curve and DCA.

**Conclusions:**

The developed radiomic nomogram model could effectively distinguish high-grade patients with BD-IPMNs preoperatively. This preoperative identification might improve treatment methods and promote personalized therapy in patients with BD-IPMNs.

**Supplementary Information:**

The online version contains supplementary material available at 10.1186/s40644-021-00395-6.

## Introduction

The pancreatic ductal system mucinous epithelium can develop mucin-producing tumors, such as intraductal papillary mucinous neoplasms (IPMNs) of the pancreas. IPMNs represent approximately 21–33% of cystic neoplasms and is one of the precursors of pancreatic cancer that is identifiable radiographically [1–3]. In the past two decades, we found that the detection rate and incidence of IPMNs have increased significantly as a result of the use of advanced diagnostic imaging technology [1, 4, 5]. When a patients is suspected of having an IPMN, determining the grade of malignancy for an individual patient permits decisions regarding whether surgery or surveillance is appropriate to be made. However, although clinicians are experienced at diagnosing and treating IPMNs, it is still challenging to distinguish high-grade IPMNs (i.e., high-grade dysplasia (HGD) to invasive carcinoma) from low-grade IPMNs (i.e., low-grade dysplasia (LGD) to intermediate-grade dysplasia (IGD)) before surgery.

Currently, laboratory tests, endoscopy, cytology, and imaging technologies play the main roles in differentiating between high-grade and low-grade IPMNs. The International Association of pancreatic diseases (IAP) recommends active surgical treatment for IPMNs of the main duct type and mixed type, while for IPMNs of the branch duct type, according to the revised 2017 international consensus guidelines, surgery is recommended for tumors with indicative features, such as mural nodules, cyst size > 3 cm, main pancreatic duct (MPD) size > 5 mm, rapid cyst growth (≥ 5 mm over 2 years), and increased serum carbohydrate antigen (CA19–9 > 37 ng/ml) levels [6, 7]. The IAP guidelines noted that the mean prevalence of invasive malignancy in BD-IPMNs was 17.7% (1–37%) [8]. In this setting, a considerable number of patients with benign lesions received unnecessary invasive surgery, and the existing grade assessment system, with unsatisfactory specificity and positive predictive value, remains unreliable [9, 10]. Therefore, there is an urgent need for a highly sensitive and specific preoperative prediction system to help establish individualized treatment decisions. Medical imaging produces essential information for the preoperative assessment of BD-IPMNs. It has been suggested that computed tomography (CT)-derived radiological features could assess the grade of BD-IPMNs objectively [11–13]. Radiomics is the quantitative analysis of images that comprise a large number of features combined with machine learning [14]. Radiomics has demonstrated potential utility in oncological imaging in prognosis, detection, and differential diagnosis assessments, such as in the lung [15], breast [16], prostate [17, 18] and liver [19]. However, to the best of our knowledge, there has been no previous research using magnetic resonance (MRI)-derived radiomics in the grade assessment in patients with BD-IPMNs. Some scoring systems or nomograms to predict malignancy from clinical variables have been suggested [20–22]; however, these methods had limitations, e.g., the lack of external or internal validation of their clinical efficacy.

Thus, the present study aimed to construct a predictive model that integrated clinical variables and a radiomic signature for preoperative grade assessment in patients with BD-IPMNs. This improved preoperative evaluation model could spare low-grade patients from potentially morbid surgery and permit high-grade patients to undergo resection before transformation into an invasive phenotype [23].

## Methods

### Workflow

**Fig. 1:**
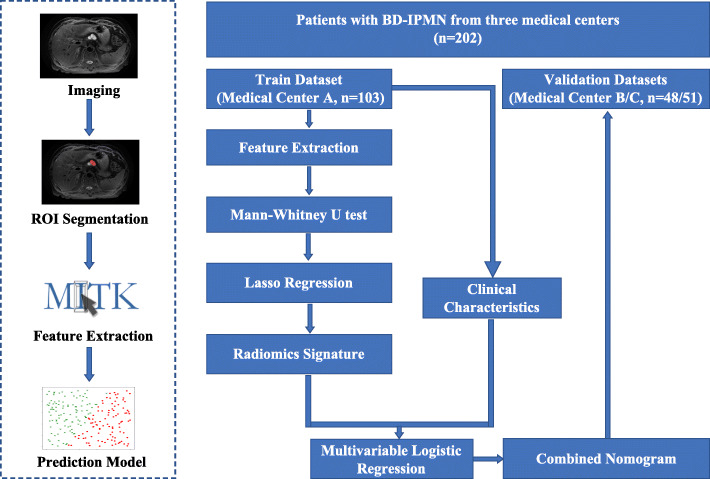
Workflow of the development of the radiomic nomogram

The research workflow is shown in Fig. 1, and comprises four parts: Acquiring the images, segmenting the region of interest (ROI), extracting features, and prediction model construction. We acquired T1-w, T2-w MRI images and CET1-w images, and radiologists outlined the tumor area manually on all image slices. Then, the quantitative radiomic features were extracted from the ROI, after which a machine learning model was established to assess the grade of BD-IPMNs.

The machine learning model-based tumor pathological grade assessment was developed and validated using three separate datasets. Medical center A (*n* = 103) data were used as the training dataset to construct the tumor pathological grade assessment model. Medical center B and C (*n* = 48 and 51) data were used as independent validation datasets to test the developed model.

After extraction of the quantitative radiomics features from the ROI, least absolute shrinkage and selection operator (LASSO) regression, Spearman correlation analysis, analysis of variance (ANOVA) tests, and Mann–Whitney U test, were applied to select the best radiomics features from the radiomic signature. Then, to construct the tumor pathological grade assessment nomogram, multivariate logistic regression was used to integrate the clinical variables and the radiomic signature.

### Patients

The Institutional Review Boards of the three centers approved this retrospective study. The provision of signed informed consent was waived. This study was conducted following the tenets of the Declaration of Helsinki. Between Mar. 2012 and Feb. 2020, patients who were reported to have BD-IPMNs on pathological assessment and who underwent an MRI scan of their pancreas before surgery were included in this study. If the branch duct-type information was not included on the pathology report, patients with BD-IPMNs were selected according to their pre-operative MRI report. Among them, 91 patients were excluded as follows: Patients lacking complete clinical data (*n* = 23), suboptimal MRI image quality caused by severe motion artifacts (*n* = 9,) and patients who did not receive an MRI scan within 3 months before surgery (*n* = 59)(Fig. S1).

A multidisciplinary team comprising, surgeons, oncologists, and radiologists assessed the patients. Clinical characteristics (e.g., sex, symptoms (abdominal pain or fatigue), age, mural nodule, cyst size, main pancreatic duct (MPD) size, CA19–9 and CEA level) were obtained through review of the clinical data by a surgeon and a radiologist with over 10 years of clinical experience. The size of the MPD was determined at the maximum dilation point of the pancreatic duct. Mural nodules were defined as any solid papillary protuberances in the cyst. The serum level of CEA and CA19–9 were measured using ELISA. The serum CA19–9 level was judged to be elevated if it was higher than the upper limit of normal (37 U/mL). The serum CEA level was judged to be elevated if it was higher than the upper limit of normal (5 U/mL). The pathological grade (LGD to invasive carcinoma) was assessed by two pathologists with over 10 years of experience in abdominal tumor diagnosis according to the 2010 World Health Organization classification [24].

### Acquisition of MRI images

Four different scanning images: preoperative T1-w, T2-w, CET1-w arterial phase, and CET1-w portal venous phase were acquired for all images. T1-w and CET1-w images were collected during breath holding and T2 images were collected by breath triggering. The details of MRI image acquisition are shown in Supplementary material I.

### Tumor segmentation and Radiomics feature extraction

To extract the features, portal venous phase imaging, arterial phase imaging, and pre-contrast T1-w, T2-w were used (Fig. S2). Radiologists manually contoured the ROIs on the MRI images. If possible, reference images were acquired using contrast-enhanced computed tomography (CT). Image standardization before ROI segmentation was conducted, which makes the thickness of the layer consistent, thus ensuring that the size and bedding of each ROI segmentation are consistent. Before ROI segmentation, the two radiologists who performed the analysis (Y.J.W. and W.Y) were blinded to the clinical outcome. The whole tumor was segmented manually using ITK-SNAP (www.itk-snap.org) on all slices of the tumor [25]. According to previously published studies, when there were multiple tumors, the tumors with the largest diameter were selected for analysis [26]. One of the radiologists drew the tumor boundary, which was verified by the other radiologist.

The MITK software (Medical Imaging Interaction Tookit 3.1.0.A, GE Healthcare) was used to extract the radiomic features from the three-dimensional ROIs, resulting in 328 features. The extracted features included: Histograms, texture parameters, RLM (run length matrix), GLCM (gray level co-occurrence matrix), and form factor parameters. Each factor’s average value was subtracted from all extracted radiometric features, and divided by the standard deviation value (Z score normalization), which eliminates the limitation imposed by each feature’s units. To standardize the different scales used to process the variables, each feature’s average value was subtracted from all the radiomic features in the training data set, and then divided by their standard deviation values, respectively. Then, using the mean and standard deviation values derived from the training dataset, the same normalization method was applied.

To construct a realistic radiomic signature that combines the most appropriate radiomic features we used the LASSO regression method, as described by Monica and Kumamaru, to select the most nonredundant and robust radiomic features [27, 28]. Supplementary material II describes the details of the LASSO method. To predict the grade assessment for each patient, the best selected radiomic features from the training cohort were recalculated using a linear combination of selected features weighted by their respective coefficients. Receiver operating characteristic (ROC) curve and area under the ROC curve (AUC) were used to evaluate the predictive accuracy of the developed radiomic signature. The reproducibility of radiomics feature extraction was assessed using intra- and inter-class correlation coefficients (ICCs). Initially, 30 ROIs were chosen randomly from each sequence. To calculate the intra-observer ICC, reader A segmentation was repeated at a 7-day interval and compared with the original segmentation. Comparing the segment extraction of reader B with that of reader A (first time) was used to calculate the inter-observer ICC. An ICC value greater than 0.8 was considered to show good consistency of feature extraction [29].

### Radiomic Nomogram construction

After univariate logistic regression, independent predictors of high-grade BD-IPMNs were selected using multivariate logistic regression analysis, and then the new radiomic nomogram was established using these independent predictors. Next, to test the calibration and recognition performance of the radiomic nomogram, the training and validation sets were used. The calibration curve displayed the performance characteristics of the multimodal radiomic nomogram models graphically. The predictive accuracy of the combined nomogram model was indicated by the degree of overlap between the diagonal in the graph and the calibration curve. The independent validation cohorts of 48 and 51 patients from the second and third medical centers were used to validate the radiomic nomogram. In the three cohorts (training and two independent validation cohorts), the clinical utility of the radiomic nomogram model was assessed using decision curve analysis (DCA). Supplementary material III shows the details of the DCA method.

### Statistical analysis

SPSS 18.0 (IBM, Armonk, NY, USA), R software (v. 3.5.1, Vienna, Austria), and MedCalc software v. 15.2.2 (https://www.medcalc.org/) were used to perform all the statistical analyses. To compare continuous variables, Mann–Whitney U-tests and the independent-sample t-test were performed when appropriate. To compare categorical variables between groups, Fisher’s exact test or a chi-squared test was used. To assess the impact of variations between intra- and inter-readers in the extracted radiomics features, intra- and inter-class correlation coefficients (ICC) were determined. In the ROC analysis, the best threshold was determined using the Youden Index. For the developed logistic regression models, the goodness of fit was examined using the Hosmer-Lemeshow test. The “glmnet” package was used to carry out the LASSO regression analysis. The “pROC” package was used to produce the ROC plots. The “rms” package was used to plot the nomogram and calibration curve. For DCA, the “rmda” package was used. Statistical significance was accepted at *P* <  0.05.

## Results

### Patients characteristics

Among the three medical centers, we recruited 202 patients diagnosed with BD-IPMNs. The training cohort comprised patients from the first medical center (*n* = 103). The external independent validation cohorts comprised the patients from the second and third medical centers (*n* = 48 and 51). The characteristics of the patients in the training and validation datasets were evenly distributed (Table S1). There were no statistically significant differences in BD-IPMN pathological grade assessment and clinical characteristics (sex, age, symptoms, mural nodule, cyst size, MPD size, CA19–9, and CEA) between the training and validation datasets. Pathologically, high-grade BD-IPMNs were detected in 24.8% patients. The detailed distribution of clinical characteristics in the low-grade and high-grade groups is summarized in Table 1. In the training and validation datasets, we noted significant differences for the cyst size, mural nodule, MPD size, and CA19–9 between the low grade and high grade groups.

**Table 1:** Characteristics of patients with IPMNs in the low-grade group and high-grade group

Characteristics	Training set (*n* = 103)			External Validation set 1 (*n* = 48)			External Validation set 2 (*n* = 51)		
	Low grade (*n* = 77)	High grade (*n* = 26)	*P*-value	Low grade (*n* = 38)	High grade (*n* = 10)	P-value	Low grade (*n* = 37)	High grade (*n* = 14)	P-value
Gender			0.683			0.854			0.792
Male	53	19		23	7		27	9	
Female	24	7		15	3		10	5	
Age (year, range)	46 ~ 83	49 ~ 79	0.185	47–79	51–80	0.061	48–79	46–79	0.647
Symptom			0.122			0.336			0.107
Yes	31	15		14	6		12	8	
No	46	11		24	4		25	6	
Largest cyst size (cm)			0.042			0.044			0.020
> 3	27	15		11	7		13	10	
≤ 3	50	11		27	3		24	4	
Size of MPD (cm)			0.005			0.047			0.002
No dilatation	28	3		11	1		12	1	
≤ 5	32	2		15	1		16	2	
5 ~ 10	12	13		10	6		7	5	
≥ 10	5	8		2	2		2	6	
Enhancing mural nodule			0.027			0.017			0.014
Yes	18	12		9	7		10	9	
No	59	14		29	3		27	5	
Thickened and enhancing cyst walls			0.116			0.278			0.247
Yes	20	11		12	5		12	7	
No	57	15		26	5		25	7	
Abrupt change in caliber of pancreatic duct with distal pancreatic atrophy			0.694			0.569			0.644
Yes	15	6		6	3		9	5	
No	62	20		32	7		28	9	
CA19–9, kU/L			< 0.001			0.001			< 0.001
Normal	65	10		31	3		33	5	
Elevated	12	16		7	7		4	9	
CEA, ng/mL			0.978			0.215			0.714
Normal	62	21		32	6		30	10	
Elevated	15	5		6	4		7	4	
Indication for surgery			0.034			0.040			0.013
High risk factors	14	10		7	5		6	7	
No high risk factors	63	16		31	5		31	7	

*MPD* Main pancreatic duct, *CA 19–9* Carbohydrate antigen, *CEA* Carcinoembryonic antigen

### Selection of Radiomics features and construction of the Radiomics signature

**Fig. 2:**
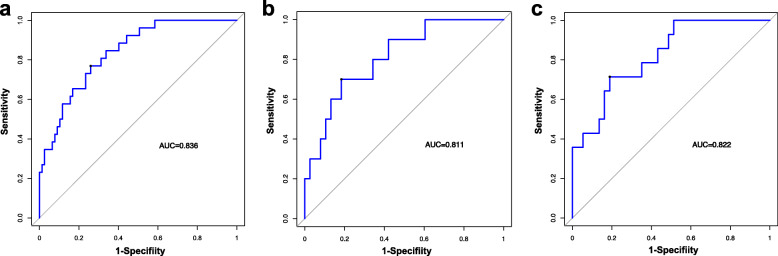
Accuracy evaluation of the radiomic signature. The radiomic signature predicted high-grade of BD-IPMNs in the training cohort (**a**) (AUC = 0.836), external validation cohort 1 (**b**) (AUC = 0.811) and external validation cohort 2(**c**) (AUC = 0822)

**Fig. 3:**
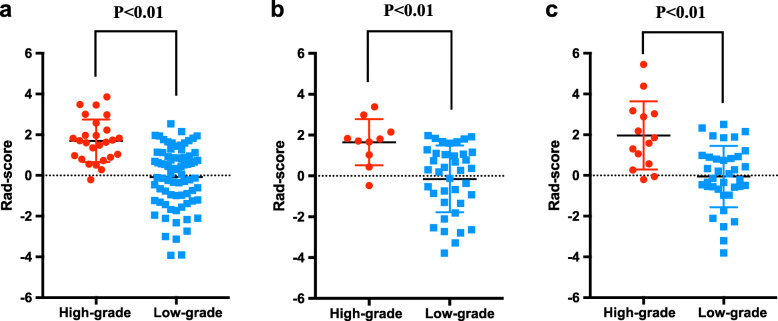
Rad-score prediction of grade of BD-IPMNs. The rad-score depicted using score dot diagrams in **a** the training cohort, **b** external validation cohort 1, and **c** external validation cohort 2. Red indicates a high grade of BD-IPMNs, blue represents a low grade of BD-IPMNs. A high score indicates a high likelihood of high-grade BD-IPMNs

In the training dataset, 334 statistically significant features (*P* <  0.05) between the low-grade and high-grade groups were identified from 1312 texture features, from which 51 features were identified by Spearman correlation analysis. To construct the radiomics signature, LASSO was used to select the nine most valuable texture features (Fig. S3), including five GLCM features, two histogram features, one texture parameter feature, and one form factor feature. The LASSO regression method derived the coefficient for each selected feature**.** Then the radiomic features classification and the calculation of texture features after dimension reduction are carried out (Table S2 and Fig. S4). Supplementary material II shows the details of the selected features and the formula used to calculate the radiomic signature. The developed radiomic signature model produced a good result when predicting the histological grade (LGD/IGD vs. HGD/associated invasive carcinoma), resulting in an AUC of 0.836 in the training set (95% confidence interval (CI), 0.750–0.901), 0.811 in validation set 1 (95% CI, 0.671–0.909), and 0.822 in validation set 2 (95% CI, 0.690–0.915). Figure 2a–c shows the ROC curves of the radiomic signature based on the three datasets. Next, we determined the quantitative scores of the radiomic signature for each patient with respect to the classification of grade assessment of BD-IPMNs, to show the effectiveness of the radiomic signature model at the individual level (Fig. 3a–c), the percentage of patients in the low grade category whose rad scores overlap with high grade category were 58.4% in the training set, 60.5% in validation set 1 and 51.4% in validation set 2. Table 2 shows the details of the performance evaluation of the radiomic signature.

**Table 2:** Univariate and multivariate logistic regression analysis of the radiomic signature and preoperative clinical parameters

Characteristics	Univariate analysis			Multivariate analysis		
	OR	95% CI	P	OR	95% CI	P
Radiomics signature	3.616	1.977 ~ 6.612	< 0.001 *	3.321	1.646 ~ 6.701	0.001 *
Age	0.963	0.910 ~ 1.018	0.185			
Symptom	2.023	0.821 ~ 4.985	0.122			
Sex	0.814	0.302 ~ 2.193	0.683			
MPD size	3.355	1.912 ~ 5.887	< 0.001 *	2.878	1.330 ~ 6.227	0.007*
Largest cyst size	2.525	1.019 ~ 6.261	0.042 *	0.413	0.088 ~ 1.929	0.261
Enhancing mural nodule	2.810	1.104 ~ 7.152	0.027 *	0.943	0.174 ~ 5.103	0.945
CA19–9 > 37kU/L	8.667	3.183 ~ 23.599	< 0.001 *	8.799	1.793 ~ 43.190	0.007*
CEA > 5 ng/mL	0.984	0.319 ~ 3.036	0.978			

*MPD* Main pancreatic duct, *CEA* Carcinoembryonic antigen, *CI* Confidence internal

Significant parameters with *P* < 0.05 in the univariate analysis were included in the multivariate logistic regression analysis

### Construction of the combined Nomogram

**Fig. 4:**
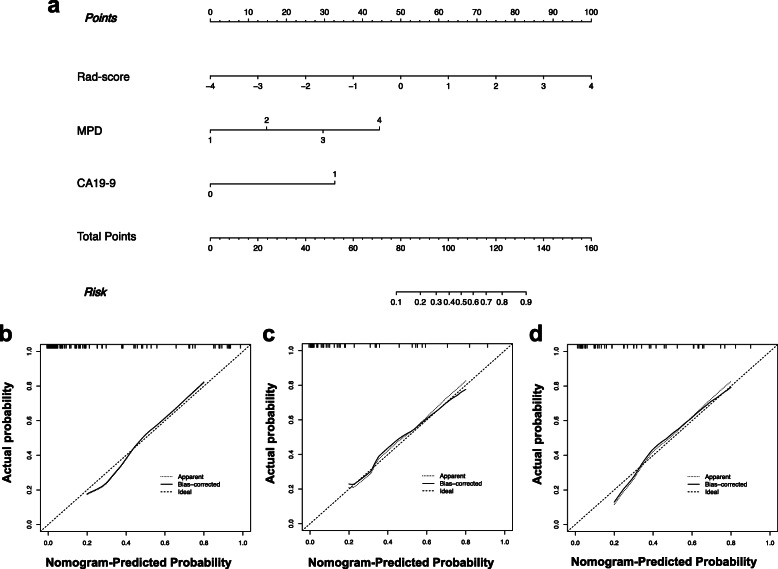
Radiomics nomogram **a** to predict high-grade BD-IPMNs. The radiomic nomogram was constructed using the rad-score, the CA19–9 level, and the size of the MPD in the data from the training cohort. Calibration for high-grade BD-IPMNs in **b** the training cohort, **c** external validation cohort 1, and **d** external validation cohort 2. The optimal nomogram is indicated by the dotted line reference line. The performance of the radiomics nomogram in high-grade prediction is indicated by the dashed line, and bias in the nomogram is corrected using the solid line

In the univariate analysis of the training cohort, the low- and high-grade groups showed significant differences for the CA19–9 level, largest cyst size, size of the MPD, mural nodule, and the radiomic signature. The AUC value for all the significant variables in the univariate analysis showed in Table 3. Multivariate logistic analysis showed that the size of the MPD (odds ratio (OR): 4.263, 95% CI: 1.762–10.3113, *P* = 0.001), the CA19–9 level (OR: 8.402, 95% CI: 1.622–43.524, *P* = 0.011), and the radiomic signature (OR: 3.434, 95% CI: 1.638–7.197, *P* = 0.001) were independent parameters of high-grade of BD-IPMN. Therefore, a radiomics nomogram model incorporating the developed radiomics signature with the size of the MPD and the CA19–9 level was constructed. A weighted number of points was assigned to each factor. The nomogram was then used to calculate the total point score of each patient, which was analyzed for its correlation with the estimated probability of high-grade of BD-IPMNs (Fig. 4a).

**Table 3:** AUC values for all the significant variables in univariate analysis of three groups

Characteristics	Training set		External Validation set 1		External Validation set 2	
	AUC	95% CI	AUC	95% CI	AUC	95% CI
Radiomics signature	0.836	0.754 ~ 0.917	0.811	0.669 ~ 0.952	0.822	0.701 ~ 0.943
MPD size	0.791	0.683 ~ 0.899	0.747	0.572 ~ 0.923	0.793	0.647 ~ 0.939
Largest cyst size	0.613	0.486 ~ .0740	0.705	0.519 ~ 0.891	0.681	0.516 ~ 0.846
Enhancing mural nodule	0.614	0.484 ~ 0.744	0.732	0.548 ~ 0.915	0.686	0.517 ~ 0.855
CA19–9 > 37kU/L	0.730	0.608 ~ 0.852	0.758	0.576 ~ 0.940	0.767	0.604 ~ 0.931

*MPD* Main pancreatic duct, *AUC* Area under the receiver operating characteristic (ROC) curve, *CI* Confidence internal

### Radiomic Nomogram performance evaluation

**Fig. 5:**
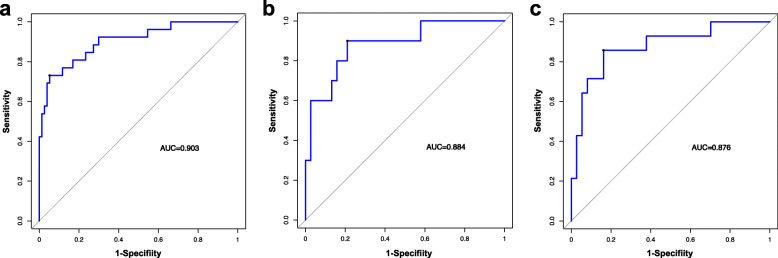
Accuracy evaluation of the radiomic nomogram. The accuracy of the radiomic nomogram to predict high-grade of BD-IPMNs was evaluated in the training cohort (**a**) (AUC = 0.903), external validation cohort 1 (**b**) (AUC = 0.884), and external validation cohort 2 (**c**) (AUC = 0.876)

ROC analysis was used to confirm the utility of the combined nomogram, resulting in an AUC of 0.903 (95% CI, 0.828–0.952), a sensitivity of 0.734, and a specificity of 0.948 for the training set; an AUC of 0.884 (95% CI, 0.759–0.958), a sensitivity of 0.900, and a specificity of 0.790 for validation set 1; and an AUC of 0.876 (95% CI, 0.754–0.952), a sensitivity of 0.857, and a specificity of 0.837 for validation set 2 (Fig. 5a–c). The AUC values showed that the combined nomogram performed well in the assessment of tumor grade. The calibration curve showed that there was sufficient consistency between the nomogram-estimated grade and the actual observed in the three cohorts (Fig. 4b-d). Table 4 shows the details of the radiomic nomogram’s performance.

**Table 4:** Predictive performance of the radiomic signature and radiomic Nomogram

Model	Radiomic signature			Radiomic nomogram		
	Specificity	Sensitivity	AUC (95% CI)	Specificity	Sensitivity	AUC (95% CI)
Training set	0.740	0.769	0.836 (0.750 ~ 0.901)	0.948	0.734	0.903 (0.828 ~ 0.952)
Validation set 1	0.816	0.700	0.811 (0.671 ~ 0.909)	0.790	0.900	0.884 (0.759 ~ 0.958)
Validation set 2	0.812	0.714	0.822 (0.690 ~ 0.915)	0.838	0.857	0.876 (0.754 ~ 0.952)

*AUC* Area under the receiver operating characteristic (ROC) curve, *CI* Confidence interval

**Fig. 6:**
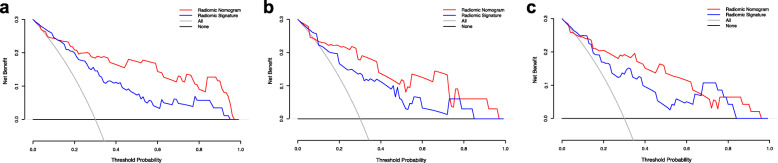
Clinical utility evaluation of the radiomic signature and the radiomic nomogram using DCA curves. Evaluation in the training cohort (**a**), external validation cohort 1 (**b**), and external validation cohort 2 (**c**). The y-axis shows the net benefit. The x-axis shows the threshold probability. The highest net benefit was gained using the radiomic nomogram (red line) compared with the radiomic signature (blue line), the treat-all strategy (gray line), and the treat-none strategy (horizontal black line)

DCA was used to reveal the utility of the combined nomogram for clinical decision making. The clinical utility of the corresponding strategies was demonstrated by the area under the decision curve (Fig. 6a–c). The area occupied by the combined nomogram (red) was larger than that of the radiomic signature (blue) alone, and was larger than those of the “all “(gray) or “none” (black) strategies in the training and the validation sets.

## Discussion

In the present study, we constructed a combined nomogram and investigated its ability to predict tumor pathological grade preoperatively in patients with BD-IPMNs. The radiomic signature was based on nine features, and was considered an effective method of preoperative tumor grade assessment, representing a non-invasive imaging biomarker. Combining the radiomics signature with clinical variables (CA19–9 and the MPD size), as a combined nomogram model, significantly improved the predictive performance. The repeatability and reliability of the developed prediction model was confirmed using independent datasets from other institutions.

Preoperative grade assessment of patients with BD-IPMNs is clinically important. According to the current consensus, most of the excised BD-IPMNs are low-grade diseases. The average incidence of invasive malignant tumors in BD-IPMNs is 17.7% (1–37%) [8]; therefore, although many patients might have dangerous signs, such as mural nodules and dilation of the main pancreatic duct, they can still be followed up for a long time without selective surgery. However, currently, the performance of the existing discrimination system is unsatisfactory, and most studies recommend immediate resection for patients with obstructive jaundice, enhanced mural nodules, and main duct dilation > 10 mm [30]. Similarly, there are no biomarkers to predict high-grade BD-IPMNs. Therefore, an advanced discriminative method with high sensitivity and specificity would provide valuable information to determine clinical strategies, resulting in patients with low-grade IPMNs avoiding unnecessary surgery for a benign disease. Of note, the goal in stratifying patients is to identify those that are at risk of developing or harboring invasive malignancy, and so even if patients with “benign” disease undergo resection this may be of treatment benefit so that they do not develop future carcinoma.

In the present study, we analyzed the predictive power of quantitative MRI features to assess the grade of BD-IPMNs. The results showed the potential value of radiomic features. We analyzed both high and low-order radiomic features identified in previous studies. The histogram parameter (low-order feature) is associated to single pixel characteristics, describing the distribution of voxel intensity via common and basic measures [31]. Hoffman et al. performed preoperative MRI texture analysis [32] and showed that intensity histogram-based statistical features and entropy from MRI images could predict the malignancy of BD-IPMNs. High order radiomic features, including GLCM features, mainly assess the spatial relationships among local neighboring pixels [33, 34]. Hanania et al. [13] identified 14 imaging biomarkers within GLCM features that predicted the histopathological grade within cyst contours. Tobaly et al. [35] developed a radiomic model mostly based on high order CT radiomic features, which showed high diagnostic performance in differentiating benign from malignant IPMNs. Our results showed that most of the selected features were high-order features, which was consistent with the results presented by previous studies. Therefore, we hypothesized that the tumor biology and heterogeneity were better represented by high-order features. Previous studies recognized the value of assessing pathological features among radiomic features, such as in nonfunctional pancreatic neuroendocrine tumors, soft-tissue masses, and rectal cancer [36–38]. However, it remains challenging to associate a single radiomic feature with the complex biological processes of tumors. Therefore, it is common to construct a multi-factor panel to estimate the results in a radiomic background. Multi-factor based radiomic methods are usually more suitable to describe the complex heterogeneity of BD-IPMNs. The results presented here indicated that our radiomic signature could satisfactorily discriminate low-grade and high-grade of BD-IPMNs in the patients in the training and validation cohorts.

According to previous studies of IPMNs, the symptoms, obstructive jaundice, presence of mural nodules, cyst size, age, and sex were also associated with pathology grade assessment [21, 39]. However, we failed to confirm these results in the present study. In addition, parameters like pain could be subjectively reported according to the different sensitivities of patients to tumor-related abdominal pain, which might also be associated with tumor infiltration into the viscera, blood vessels, or peripheral nerves. We observed that age and sex were not related to grade, which might be related to populations from different ethnicities. Obstructive jaundice was rare in the patients in our study, which could be related to the limited sample size.

The radiomic nomogram constructed in our study incorporated clinical factors (the size of MPD and CA19–9 levels), which might be useful to identify those patients at risk of malignancy and to select the best treatment.

The CA19–9 level is useful for the differential diagnosis of pancreatic carcinoma and benign pancreatic diseases, and increased CA19–9 levels are an independent predictor of malignant BD-IPMNs [20, 40–42]. However, in the present study, CA19–9 alone achieved an AUC of 0.762 to discriminate high-grade in patients training set. Thus, CA19–9 alone is not sufficient to evaluate the high-grade of BD-IPMNs accurately. Previous studies showed that main duct dilatation was a significant predictor of malignancy and suggested that the patients with main duct dilatation over 10 mm should undergo resection without further testing or calculation [39]. In the present study, we divided the MPD dilation into four degrees according to previous studies [10, 39], and found that patients with a larger MPD dilation were more likely to have a high-grade of BD-IPMNs.

The DCA curve showed that the radiomic nomogram was superior to the radiomic signature over a large threshold probability range, which indicated that the clinical parameters increased the incremental value for BD-IPMN grade assessment in the training and validation sets.

The established grade assessment model has obvious advantages. For clinicians seeking low-cost techniques to improve patient management, quantitative image analysis is attractive, because it comprises a noninvasive disease assessment at multiple time points and tumor sites compared with conventional diagnostic imaging. In the current study, the results showed that the AUC of the model was further improved to 0.903 by combining the nomogram with clinical characteristics and the radiomic signature, suggesting that advanced imaging technology should be considered together with well-known clinical factors. In addition, the pre-operative data used to establish the grade assessment model is easy to obtain, with less additional costs and provides good results.

The present study had certain limitations. First, because of the retrospective design of our study, there may be selection bias. Second, this study involved a relatively small sample, and the low-grade and high-grade groups were not further separated into patients with low and intermediate-grade dysplasia or high-grade dysplasia and patients with associated invasive carcinoma. However, our cohort was sufficient to build a reliable model. Third, the area of potential peritumoral infiltration were not included in this study, which may result in underestimation of tumor area. In future, studies are still needed for detailed analysis of features extracted from peritumoral area. However, as we focus on the visible tumor area, the results are still reliable. Our study had another limitation: Tumor area segmentation has to be performed manually by radiologists. Automatic segmentation will be more convenient and should become the standard. In the future, we will construct a fully automatic prediction model incorporating automatic segmentation of the pancreas and cyst areas.

## Conclusions

The preoperative pathological grade of BD-IPMNs could be predicted effectively using the developed nomogram model combining the radiomic signature and tumor clinical characteristics. The predictive nomogram model represents an accurate and noninvasive assessment method for patients with BD-IPMNs before surgery, which will help clinicians to alter the treatment protocol for each patient and thus obtain improved clinical outcomes in the future.

## Supplementary Information

**Additional file 1.** The Supplementary Material for this article was available in additional file 1.

## Data Availability

The datasets used and/or analyzed during the current study are available from the corresponding author on reasonable request.

## Abbreviations

BD-IPMNs: Branching type intraductal papillary mucinous neoplasms
MPD: Main pancreatic duct
LGD: Low-grade dysplasia
IGD: Intermediate-grade dysplasia
HGD: High-grade dysplasia
