# Supplementary material for: Radiomic nomogram based on MRI to predict grade of branching type intraductal papillary mucinous neoplasms of the pancreas: a multicenter study
Source: Cancer Imaging. 2021 Mar 9;21:26. doi: 10.1186/s40644-021-00395-6 (PMC7942000; doi:10.1186/s40644-021-00395-6)
Supplement: Supplementary file 1 — Additional file 1. The Supplementary Material for this article was available in additional file 1. [file 40644_2021_395_MOESM1_ESM.docx]

**Supplementary material**

**I. MRI image acquisition**

In center A, preoperative MR imaging was performed with a 3.0 T system (Signa HDxT, GE Medical System, Waukesha, WI, USA). The parameters were as follows: T1-w: repetition time (TR) = 4ms, echo time (TE) = 1.7ms, the slice thickness = 3mm, matrix = 288*180, and field of view(FOV) = 38cm*38cm; T2WI: TR =6000~8000ms, TE=91 ms, slice thickness =6 mm, matrix = 320*224, and FOV = 38cm*38 cm; Contrast-enhanced imaging was performed following the intravenous injection of 0.1 mmol/kg contrast medium (Gadodiamide, GE Healthcare, Waukesha, WI, USA), rate= 2 mL/s. The arterial phase and portal venous phase (20 and 60 sec post-injection) were collected respectively. TR=3.2 ms, TE=1.5 ms, slice thickness = 2.5 mm, matrix = 320*256, and field of view (FOV) = 38 × 38 cm.

In center B, the examination was performed with a 3.0-T system (Discovery MR750; GE Healthcare, Waukesha, WI, USA). The parameters were as follows: T1-w: TR= 3.9 ms, TE=1.2 ms, the slice thickness = 3 mm; matrix= 256 x 256, and FOV= 38cm*38cm. T2WI: TR= 6000~8000 ms, TE= 91 ms, the slice thickness = 4 mm; matrix= 256 x 256, and FOV= 38cm*38cm. Contrast-enhanced imaging was performed following the intravenous injection of 0.1 mmol/kg contrast medium (Gadodiamide, GE Healthcare, Waukesha, WI, USA), rate= 2 mL/s. The imaging delay time for arterial phase and portal venous phase was 20 s and 50 s, respectively. TR=3.8ms, TE=1.7ms, FOV=380x300mm, matrix=320*224, slice thickness=3.2mm.

In center C, the examination was performed with a 3.0-T system (Discovery MR750; GE Healthcare, Waukesha, WI, USA). The parameters were as follows: T1-w: TR= 3.8 ms, TE=2.0 ms, the slice thickness = 4 mm; matrix= 260 x 224, and FOV= 34cm*34cm. T2WI: TR= 6000~8000 ms, TE= 68 ms, the slice thickness = 4 mm; matrix= 288 x 288, and FOV= 40cm*40cm. Contrast-enhanced imaging was performed following the intravenous injection of 0.1 mmol/kg contrast medium (Gadodiamide, GE Healthcare, Waukesha, WI, USA), rate= 2 mL/s. The arterial phase and portal venous phase (20 and 50 sec post-injection) were collected respectively. TR=3.5 ms, TE=2.0 ms, slice thickness = 3 mm, field of view (FOV) = 38 × 38 cm , and matrix = 300*256.

**II. Radiomics Features Extraction**

**2.1 Dimension reduction of radiomics features**

First, the extracted texture features were standardized (z-score), which removed the unit limits of the data of each feature so that the indexes of different units or orders could be compared and weighted. Then, z-score normalization was used to make the image intensities have the properties of a standard normal distribution with $\mu=1$ and $\sigma=0$, where $\sigma$ was the mean value of the images, and $\mu$ was the standard deviation. The normalized values (also called z scores) of the image intensities (x) were calculated as follows:

$$z=\frac{\chi-\mu}{\sigma}$$

Then, feature dimensionality reduction was performed as follows. Analysis of variance (ANOVA) and Mann-Whitney U test (MW) dimensionality reduction were performed, and then the correlation value was calculated to reduce data redundancy. The software calculated the paired correlation between each pair of features. If the Spearman correlation coefficient was greater than 0.9, it was believed to approach a linear relationship. In other words, the two features could express each other, which had no effect on the result; however, the coefficient was different when fitting. In this study, the software automatically removed the feature of the latter when the two features were relatively relevant. All the above steps were carried out using the AK software. Then, the least absolute shrinkage and selection operator (LASSO) was used to select the most useful predictive radiomic features.

**2.2 The LASSO algorithm**

LASSO is a powerful algorithm for regression analysis with high dimensional predictors. In the present study, the LASSO algorithm was combined with the logistic regression model for model development. We used the LASSO logistic regression model to select the most important predictive features and construct a radiomics signature in the training set. This algorithm minimizes a log partial likelihood subject to the sum of the absolute values of the parameters bounded by a constant:

$\hat{\beta}=arg\min\mathcal{l}\left( \beta\right)$ subject to $\sum\left| \beta_{i} \right|\leq t$ where $\hat{\beta}$ is the obtained parameter, $\mathcal{l}\left( \beta\right)$ is the log partial likelihood of the logistic regression model, and $t>0$is a constant.

The LASSO algorithm shrinks some coefficients and reduces others to exactly 0 via the absolute constraint. Thus, LASSO is an outstanding method for feature selection that retains the good features of both subset selection and ridge regression. In this study, the constant t was set as 0.015, and LASSO selected nonzero coefficients `1$\hat{\beta}$, and a formula was generated using a linear combination of selected features that were weighted by their respective LASSO coefficients. The “glmnet” package in the R statistical software version 3.4.1 was used for LASSO logistic regression model analysis; the specific formula is as follows.

Radiomics signature calculation formula:

Rad-score= -1.2811+0.7426*T1_ClusterProminence_AllDirection_offset1

-0.60747*T1_GLCMEnergy_angle0_offset1

-0.29509*T2_histogramEnergy

+0.64849*T2_GLCMEntropy_AllDirection_offset1

-0.48698*CET1A_InverseDifferenceMoment_ AllDirection_offset1

+0.91879* CET1A_Correlation_ AllDirection_offset7

+1.12872*CET1P_Variance

+1.09587*CET1P_GLCMEntropy_AllDirection_offset7

+1.64742*CET1P_VolumeCC

Fig. S4 shows the flowchart of radiomic features dimension reduction. Supplementary Table S2 shows the radiomic features classification and calculation formula of texture features after dimension reduction.

**2.3 Detailed descriptions of features**

**2.3.1 Information on the features**

Three hundred and twenty-eight radiomic features from each phase were calculated using the AK software. Radiomic features included Histogram, Formfactor, Gray-Level Co-occurrence Matrix (GLCM), and Run length matrix (RLM). The details are described in the table below.


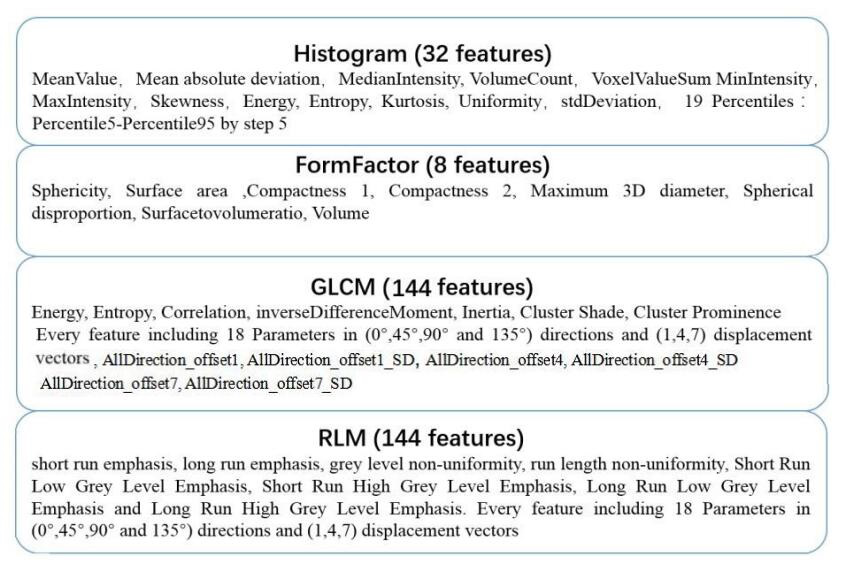


**2.4 Details of the GLCM**

**Co-occurrence matrices**

In the Gray Level Co-occurrence Matrix (GLCM), $P\left( i,\left. j \right|\theta,d \right)$ represents the joint probability of certain sets of pixels having certain gray-level values. It calculates how many times a pixel with gray-level $i$occurs jointly with another pixel having a gray value j, by varying the displacement vector$d$between each pair of pixels.

The advantage of the co-occurrence matrix calculations is that the co-occurring pairs of pixels can be spatially related in various orientations with reference to distance and angular spatial relationships, considering the relationship between two pixels at a time. As a result, the combination of gray levels and their positions are exhibited. Therefore, it is defined as “A two-dimensional histogram of gray levels for pair of pixels, which are separated by a fixed spatial relationship”. However, the matrix is sensitive to rotation. Changes in different offsets define pixel relationships by varying directions.

The rotation angle of an offset:0°,45°,90°,135°and displacement vectors (distance to the neighbor pixel: 1, 2, 3 ...), result in different co-occurrence distributions from the same image of reference. GLCM of an image is computed using displacement vector d defined by its radius, (distance or count to the next adjacent neighbor is preferably equal to one) and rotational angles.


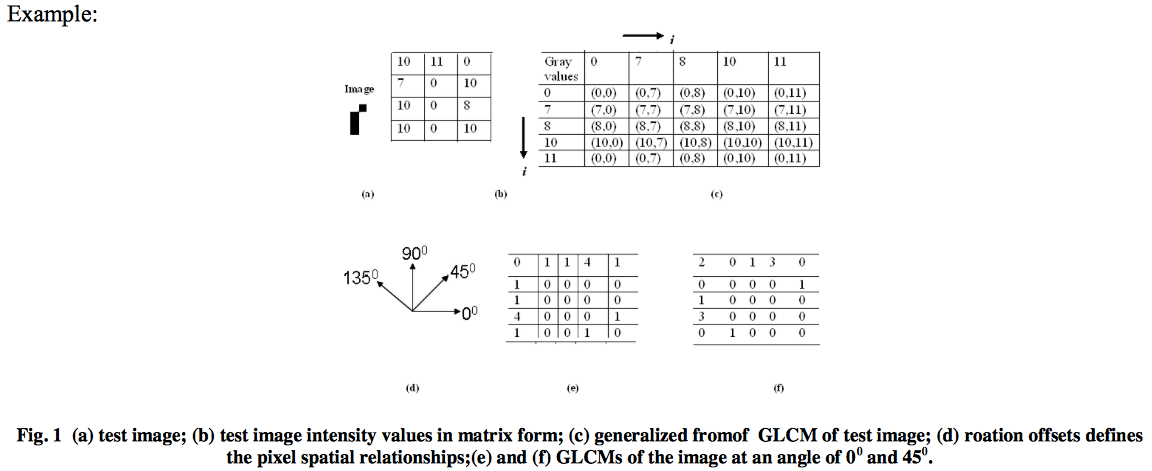


**III. Definition of net benefit in the decision curve analysis.**

Decision curve analysis (DCA) was used to assess the clinical utility of the radiomic nomogram model in the three cohorts. The "true" positive and weighted false-positive rates were calculated across different threshold probabilities in the validation set to determine the net benefit. Specifically, the weighting factor was defined as the specific value of the threshold probability divided by 1 minus the threshold probability. A higher true-positive rate and a relatively low false-positive rate were suggested by a high net benefit. Plotting the net benefit against the threshold probability across the range of 0 to 0.8 generated the decision curve.

The net benefit was defined by the following equation:

Net Benefit = $TPR*\omega-\frac{P_{t}}{1-P_{t}}*FPR*\left( 1-\omega\right)$

𝑃𝑡 is the “threshold possibility” to stratify the patients into high-risk or low-risk groups. Patients with a probability of having high-grade dysplasia /associated invasive carcinoma IPMNs higher than 𝑃𝑡 are high-risk patients. These patients would be recommended for aggressive intervention(s), while others (low-risk) would be referred to relatively mild treatment(s). TPR is the true positive rate, defined as the proportion of high-risk patients in the patients having high-grade dysplasia /associated invasive carcinoma IPMNs. FPR is the false positive rate, defined as the proportion of high-risk patients in the patients having LGD/IGD IPMNs. 𝜔,is the prevalence of having HGD/associated invasive carcinoma IPMNs, calculated by dividing the total patients number by the number of patients with HGD /associated invasive carcinoma IPMNs. In the condition of “treat none”, no patient is classified as high risk, both the TPR and FPR are zero, so the Net Benefit is zero. In the condition of “treat everyone”, all patients are classified as high risk (TPR=FPR=1), so the Net Benefit is calculated as

$${Net Benefit}_{Treat Everyones}=\omega-\frac{P_{t}}{1-P_{t}}*\left( 1-\omega\right)=\frac{1-\omega}{P_{t}-1}+1$$

, which is a monotonically decreasing curve in the figure.

**Reference:**

[1]Kerr KF, Brown MD, Zhu K, Janes H. Assessing the Clinical Impact of Risk Prediction Models With Decision Curves: Guidance for Correct Interpretation and Appropriate Use. Journal of Clinical Oncology 2016;34(21):2534-40 doi10.1200/JCO.2015.65.5654.

[2] Vickers AJ, Van Calster B, Steyerberg EW. Net benefit approaches to the evaluation of prediction models, molecular markers, and diagnostic tests. The BMJ 2016;352:i6. doi10.1136/ bmj.i6.

**Fig. S1 The recruitment pathway in this study.**

**Fig. S2 Imaging of representative patients.**

Figure T1-w (A), T2-w (B), CET1-Aetiral Phase (C), CET1-Portal Vein Phase (D) and ROIs of a 65-year male was diagnosed with BD-IPMNs in the body of the pancreas.

**Fig. S3 LASSO logistic regression to select radiomic features.**

(a)In the LASSO model, for 10-fold cross-validation, the tuning parameter ( λ) was chosen as the minimum criterion. The x-axis shows the value of log (λ) and the y-axis shows the binominal deviance in the 10-fold cross-validation. The relationship between partial likelihood deviation and log λ is drawn. Dashed vertical lines at the best values were drawn using the minimum criteria and 1-SE criteria. (b) The 51 texture features identified in the LASSO coefficient profile. Ten-fold cross-validation was used to draw the vertical line in the log λ) sequence at the selected value, and indicated nine features with non-zero coefficients.

**Fig. S4 Flowchart of radiomic features dimension reduction.**

Table S1 shows the Characteristics of the study population

| Table S1. Characteristics of the study population | | | | | | | |
| --- | --- | --- | --- | --- | --- | --- | --- |
| Characteristics | Training set (n=103) | | External Validation set 1(n = 48) | | External Validation set 2(n = 51) | | P value |
|  | n | % | N | % | N | % |
| Gender |  |  |  |  |  |  | 0.609 |
| Male | 72 | 69.9% | 30 | 62.5% | 36 | 70.6% |  |
| Female | 31 | 30.1% | 18 | 37.5% | 15 | 29.4% |  |
| Age (year, range) | 65 | 46–83 | 66 | 47–80 | 67 | 46–79 | 0.871 |
| Symptom |  |  |  |  |  |  | 0.805 |
| Yes | 46 | 44.7% | 20 | 41.7% | 20 | 39.2% |  |
| No | 57 | 55.3% | 28 | 58.3% | 31 | 60.8% |  |
| Largest cyst size (cm) |  |  |  |  |  |  | 0.741 |
| > 3 | 42 | 40.8% | 18 | 37.5% | 23 | 45.1% |  |
| ≤ 3 | 61 | 59.2% | 30 | 62.5% | 28 | 54.9% |  |
| Size of MPD (mm) |  |  |  |  |  |  | 0.821 |
| No dilatation | 30 | 29.1% | 12 | 25.0% | 13 | 25.5% |  |
| ≤ 5 | 33 | 32.0% | 16 | 33.3% | 18 | 35.3% |  |
| 5~10 | 27 | 26.2% | 16 | 33.3% | 12 | 23.5% |  |
| ≥ 10 | 13 | 12.6% | 4 | 8.4% | 8 | 15.7% |  |
| Mural nodule |  |  |  |  |  |  | 0.585 |
| Yes | 30 | 29.1% | 16 | 33.3% | 19 | 37.3% |  |
| No | 73 | 70.9% | 32 | 66.7% | 32 | 62.7% |  |
| CA19-9, kU/L |  |  |  |  |  |  | 0.919 |
| Normal | 75 | 72.8% | 34 | 70.8% | 38 | 74.5% |  |
| Elevated | 28 | 27.2% | 14 | 29.2% | 13 | 25.5% |  |
| CEA, ng/mL |  |  |  |  |  |  | 0.979 |
| Normal | 83 | 80.6% | 38 | 79.2% | 40 | 78.4% |  |
| Elevated | 20 | 19.4% | 10 | 20.8% | 11 | 21.6% |  |
| Pathological grade |  |  |  |  |  |  | 0.738 |
| Low grade | 77 | 74.8% | 38 | 79.2% | 37 | 72.5% |  |
| High grade | 26 | 25.2% | 10 | 20.8% | 14 | 27.5% |  |
| MPD, main pancreatic duct; CA 19-9, carbohydrate antigen; CEA, carcinoembryonic antigen; | | | | | | | |

Table S2 shows the radiomic features classification and calculation formula of texture features after dimension reduction.

| Table S2. The classification and calculation formula of nine texture features | | | |
| --- | --- | --- | --- |
| Phase | Category | Feature | Formula |
| T1 | Texture Parameters | ClusterProminence_AllDirection_offset1 | 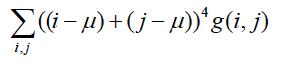 |
|  | GLCM | GLCMEnergy_angle0_offset1 |  |
| T2 | Histogram | Energy | $Energy=\sum_{i}^{N} X{(i)}^{2}$ |
|  | GLCM | GLCMEntropy_AllDirection_offset1 | $\sum_{i,j} g(i,j)\log_{2} (i,j)$ *g is a GLCM  Where i,j are the spatial coordinates of g (i,j). |
| Arterial Phase | GLCM | InverseDifferenceMoment_AllDirection_offset1 | $\sum\sum\frac{1}{1+{（i-j）}^{2}}g\left( i,j \right)$ *g is a GLCM  Where $i,j$are the spatial coordinates of $g(i,j)$ |
|  | GLCM | Correlation_AllDirection_offset7 | $-\sum_{i,j} \frac{(i-\mu)(j-\mu)g(i,j)}{\sigma^{2}}$ |
| Portal Venous Phase | Histogram | Variance | $variance=\frac{1}{N-1}\sum_{i=1}^{N} \left( X\left( i \right)-\bar{X} \right)^{2}$ |
|  | GLCM | GLCMEntropy_AllDirection_offset7 | $\sum_{i,j} g(i,j)\log_{2} (i,j)$ *g is a GLCM  Where i,j are the spatial coordinates of g (i,j). |
|  | Form Factor | VolumeCC | $V=\text{d}\times A$ |
